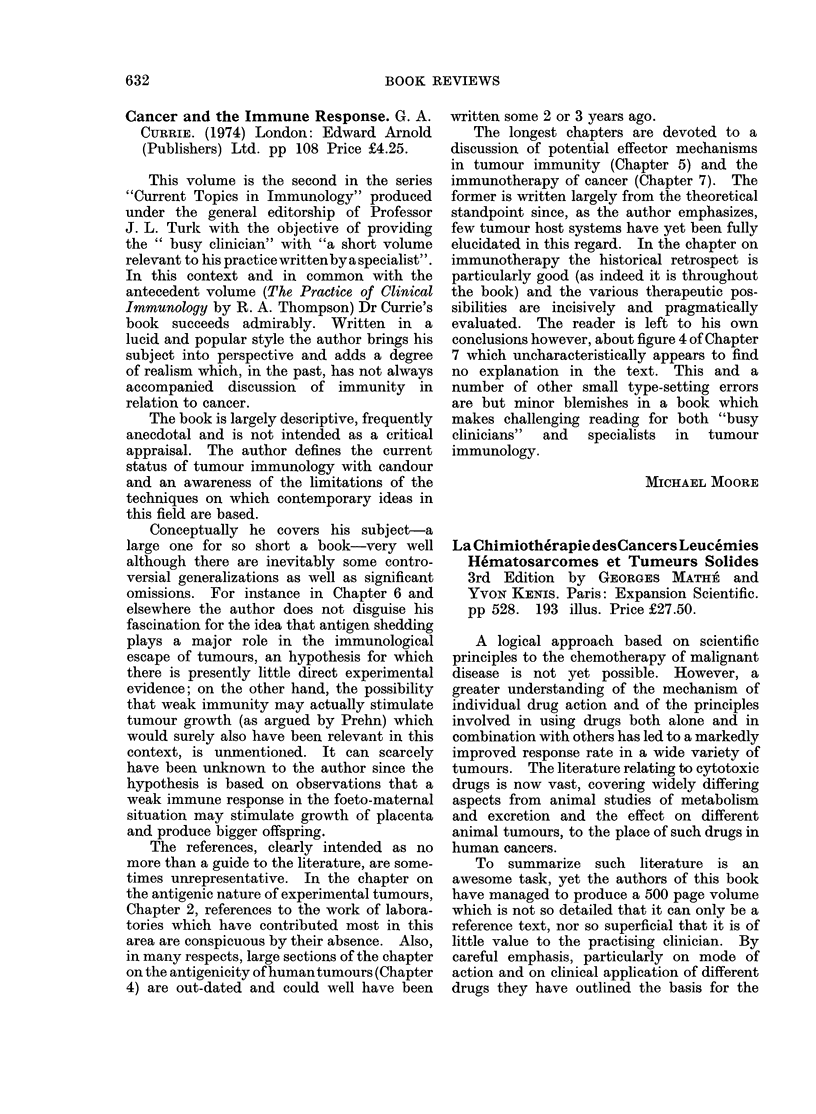# Cancer and the Immune Response

**Published:** 1975-11

**Authors:** Michael Moore


**Cancer and the Immune Response.** G. A. CURRIE. (1974) London: Edward Arnold (Publishers) Ltd. pp 108 Price £4.25.

This volume is the second in the series "Current Topics in Immunology" produced under the general editorship of Professor J. L. Turk with the objective of providing the " busy clinician" with "a short volume relevant to his practice written by a specialist". In this context and in common with the antecedent volume (*The Practice of Clinical Immunology* by R. A. Thompson) Dr Currie's book succeeds admirably. Written in a lucid and popular style the author brings his subject into perspective and adds a degree of realism which, in the past, has not always accompanied discussion of immunity in relation to cancer.

The book is largely descriptive, frequently anecdotal and is not intended as a critical appraisal. The author defines the current status of tumour immunology with candour and an awareness of the limitations of the techniques on which contemporary ideas in this field are based.

Conceptually he covers his subject—a large one for so short a book—very well although there are inevitably some controversial generalizations as well as significant omissions. For instance in Chapter 6 and elsewhere the author does not disguise his fascination for the idea that antigen shedding plays a major role in the immunological escape of tumours, an hypothesis for which there is presently little direct experimental evidence; on the other hand, the possibility that weak immunity may actually stimulate tumour growth (as argued by Prehn) which would surely also have been relevant in this context, is unmentioned. It can scarcely have been unknown to the author since the hypothesis is based on observations that a weak immune response in the foeto-maternal situation may stimulate growth of placenta and produce bigger offspring.

The references, clearly intended as no more than a guide to the literature, are sometimes unrepresentative. In the chapter on the antigenic nature of experimental tumours, Chapter 2, references to the work of laboratories which have contributed most in this area are conspicuous by their absence. Also, in many respects, large sections of the chapter on the antigenicity of human tumours (Chapter 4) are out-dated and could well have been written some 2 or 3 years ago.

The longest chapters are devoted to a discussion of potential effector mechanisms in tumour immunity (Chapter 5) and the immunotherapy of cancer (Chapter 7). The former is written largely from the theoretical standpoint since, as the author emphasizes, few tumour host systems have yet been fully elucidated in this regard. In the chapter on immunotherapy the historical retrospect is particularly good (as indeed it is throughout the book) and the various therapeutic possibilities are incisively and pragmatically evaluated. The reader is left to his own conclusions however, about figure 4 of Chapter 7 which uncharacteristically appears to find no explanation in the text. This and a number of other small type-setting errors are but minor blemishes in a book which makes challenging reading for both "busy clinicians" and specialists in tumour immunology.

MICHAEL MOORE